# Antibiotics Use in COVID-19 Patients: A Systematic Literature Review

**DOI:** 10.3390/jcm11237207

**Published:** 2022-12-04

**Authors:** Guido Granata, Francesco Schiavone, Giuseppe Pipitone, Fabrizio Taglietti, Nicola Petrosillo

**Affiliations:** 1 Clinical and Research Department for Infectious Diseases, National Institute for Infectious Diseases L. Spallanzani, IRCCS, 00149 Rome, Italy; 2 Divers and Raiders Group Command “Teseo Tesei” COMSUBIN, Medical Service, Italian Navy, 19025 Portovenere, Italy; 3 Infectious Disease Unit, ARNAS Civico-Di Cristina, Piazza Leotta, 5, 90100 Palermo, Italy; 4 Fondazione Policlinico Universitario Campus Bio-Medico, 00128 Rome, Italy

**Keywords:** COVID-19 pandemic, bacterial infections, bacterial coinfection, systematic review, antibiotics use in COVID-19 patients, antibiotics, antimicrobials, COVID-19 inpatients, COVID-19 outpatients, guidelines for the clinical management of COVID-19

## Abstract

The issue of bacterial infections in COVID-19 patients has received increasing attention among scientists. Antibiotics were widely prescribed during the early phase of the pandemic. We performed a literature review to assess the reasons, evidence and practices on the use of antibiotics in COVID-19 in- and outpatients. Published articles providing data on antibiotics use in COVID-19 patients were identified through computerized literature searches on the MEDLINE and SCOPUS databases. Searching the MEDLINE database, the following search terms were adopted: ((antibiotic) AND (COVID-19)). Searching the SCOPUS database, the following search terms were used: ((antibiotic treatment) AND (COVID-19)). The risk of bias in the included studies was not assessed. Both quantitative and qualitative information were summarized by means of textual descriptions. Five-hundred-ninety-three studies were identified, published from January 2020 to 30 October 2022. Thirty-six studies were included in this systematic review. Of the 36 included studies, 32 studies were on the use of antibiotics in COVID-19 inpatients and 4 on antibiotic use in COVID-19 outpatients. Apart from the studies identified and included in the review, the main recommendations on antibiotic treatment from 5 guidelines for the clinical management of COVID-19 were also summarized in a separate paragraph. Antibiotics should not be prescribed during COVID-19 unless there is a strong clinical suspicion of bacterial coinfection or superinfection.

## 1. Introduction

The issue of bacterial infections in COVID-19 patients has received increasing attention among scientists. Most COVID-19 deaths are the result of severe viral pneumonia, especially in the older and frail population. To date, corticosteroids are a therapeutic class that has proven beneficial in terms of mortality in COVID-19 pneumonia [1]. However, immunosuppressive treatment and hospitalization are associated with an increased risk of bacterial infection [2].

So far, bacterial coinfections during COVID-19 have been reported in percentages ranging from 7.2% to 8.5% [2,3]. To consider, based only on clinical presentation, it is difficult to distinguish between a bacterial and a viral pulmonary infection.

Strikingly, this scenario conflicts with the general practice, where antibiotics are widely prescribed to COVID-19 patients. The extensive use of broad-spectrum empirical antibiotic prescribing in COVID-19 may cause unintended consequences of antimicrobial usage, i.e., the rise of multidrug-resistant bacteria [4].

We performed a literature review with the main aim to assess the reasons, evidence and practices on the use of antibiotics in COVID-19 inpatients and outpatients.

## 2. Materials and Methods

### 2.1. Search Strategy and Article Identification

Published articles on antibiotics use in COVID-19 patients were identified through computerized literature searches using the MEDLINE (National Library of Medicine Bethesda MD) and SCOPUS database.

Searching the MEDLINE database, the following search terms were used: [(antibiotic) AND (COVID-19)]. The following filters were applied: Article type: Clinical Study, Clinical Trial, Observational Study, Randomized Controlled Trial; Language: English; Age: Adult; Publication date: from 1 January 2020 to 1 October 2022.

Searching the SCOPUS database, the following search terms were used: [(antibiotic treatment) AND (COVID-19)]. The following filters were applied: Subject area: Medicine; Document type: Article; Keyword: Outcome Assessment; Language: English; Publication date: from 2020 to 2022.

Supplementary Figure S1 describes the complete specifications of the query details used on the MEDLINE and SCOPUS databases, respectively (Figure S1).

No attempt was made to obtain information about unpublished studies. Studies published only in abstract form, correction articles, review articles and meta-analyses, case reports, editorials and clinical trial protocols were excluded from further assessment.

Apart from the studies identified and included in the review, the main recommendations on antibiotic treatment from 5 guidelines for the clinical management of COVID-19 are also summarized in a separate paragraph.

### 2.2. Eligibility Criteria

Studies including original articles and randomized clinical trials reporting data on the antibiotic use in COVID-19 patients were eligible for inclusion in our review.

### 2.3. Study Selection and Data Extraction

Eligibility assessment and extraction of data were performed independently by two investigators. Each investigator was blinded to the other investigator’s data extraction. In case of disagreement between the two reviewers, a third reviewer was consulted. Data from each study were verified for consistency and accuracy, and then entered into a standardized computerized database. The risk of bias in the included studies was not assessed. Abstracted information included: author, year of publication, country in which the study was conducted; study design, start and end date of study, healthcare/community setting, sample size; criteria for the diagnosis of severe acute respiratory syndrome coronavirus 2 (SARS-CoV-2)/bacterial infection, if applicable; proportion of patients receiving antibiotic treatment, if applicable; data on patients’ outcome, if applicable.

### 2.4. Data Synthesis

For the data syntheses, included articles were grouped into two groups: studies on antibiotic use in COVID-19 inpatients; studies on antibiotic use in COVID-19 outpatients. Both quantitative and qualitative information were summarized by means of textual descriptions.

## 3. Results

### 3.1. Studies Description

**Figure 1:**
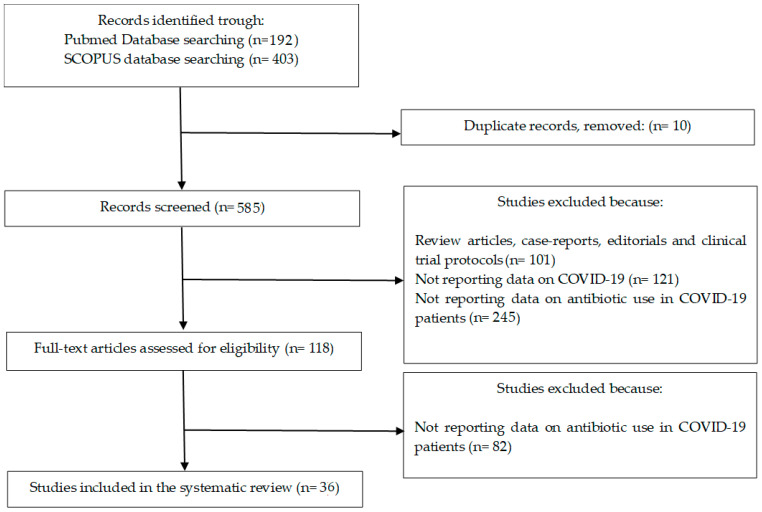
Flowchart depicting the selection process of studies included in the systematic review.

Figure 1 shows the selection process of studies included in the review. Through computerized literature searches we identified 585 studies published from January 2020 to 30 October 2022. One-hundred-one studies were excluded because they were review articles, meta-analyses, case reports, editorials and clinical trial protocols. One-hundred-twenty-one studies were excluded for not reporting data on COVID-19. Two-hundred-forty-five studies were excluded for not reporting data on antibiotic use in COVID-19 patients. From the remaining 118 studies, 36 studies were included in this systematic review (Figure 1) [5,6,7,8,9,10,11,12,13,14,15,16,17,18,19,20,21,22,23,24,25,26,27,28,29,30,31,32,33,34,35,36,37,38,39,40].

Of the 36 studies included in the systematic review, 32 studies were on the use of antibiotics in COVID-19 inpatients [5,6,7,8,9,10,11,12,13,14,15,16,17,18,19,20,21,22,23,24,25,26,27,28,29,30,31,32,33,34,35,36]; 4 reported on antibiotic use in COVID-19 outpatients [37,38,39,40].

Of these studies, 8 were clinical trials: 4 phase IIII trials evaluating antibiotic use in COVID-19 inpatients [17,18,20,24] and 4 phase III clinical trials evaluating antibiotic use in COVID-19 outpatients [37,38,39,40]. A summary description of the included clinical studies is reported in Table 1 and Table 2.

### 3.2. Use of Antibiotics in COVID-19 Patients

#### 3.2.1. Hospitalized Patients

##### Bacterial Infection and Antibiotics Use in Hospitalized COVID-19 Patients

Table 3 summarizes the findings of studies on bacterial infection and antibiotics use in hospitalized COVID-19 patients [5,6,7,8,9,10,11,12,13,14,31,32,33,34,35,36]. The majority of these studies report a low prevalence of bacterial coinfection in hospitalized COVID-19 patients.

A large observational cohort study was performed in Spain with the aim of describing the epidemiology and outcomes of bacterial coinfections and superinfections occurring in hospitalized COVID-19 patients [5]. The authors reported that among 989 COVID-19 patients, the presence of a community-acquired coinfection at COVID-19 diagnosis was uncommon (31/989, 3.1%) and mainly due to *S. pneumoniae* and *S. aureus*. Hospital-acquired bacterial superinfections, mostly caused by *P. aeruginosa* and *E. coli*, were diagnosed in 43 patients (4.7%). The reported overall mortality was 9.8% (97/989), and patients with hospital-acquired superinfections had worse outcomes (18.6%, 8/43).

Similar findings came from a large, multicenter retrospective cohort study performed in The Netherlands [6]. This study included 925 COVID-19 patients, and 12 (1.2%) of them had documented bacterial coinfections (75% pneumonia) within the first week of hospitalization. Interestingly, the authors reported that 556 of the 925 included patients (60.1%) received antibiotics before hospital admission.

Moreover, an observational cohort study performed in two hospitals in London reported that among 1396 hospitalized COVID-19 patients, 37 (2.7%) had bacterial coinfection within 48 hours of admission. The majority of the included patients (up to 98%) received empirical antibiotic treatment [7].

Ruiz-Bastián et al., performed a single-center retrospective observational study in critically ill COVID-19 patients. They reviewed bacterial isolates from respiratory samples during the first two months of the pandemic [13]. A total of 1251 respiratory samples from 1195 patients were processed. Samples from 66 patients (5.52%) were positive. In line with other studies, all the included patients received broad-spectrum antibiotics as empirical treatment. The isolated bacteria were mainly Enterobacterales followed by *S. aureus* and *P. aeruginosa* [13].

In a multicenter observational study, Giannella et al., developed a score to stratify patients at low, intermediate and high risk of bacterial coinfection to optimize antibiotic use. Among 1733 COVID-19 patients, 59.8% received antibiotics at admission. The reported rate of bacterial infections was 6.3%. The most common types of bacterial coinfection were community-acquired pneumonia, urinary tract infection and bloodstream infection [33].

Of note, the remaining studies reporting a higher prevalence of bacterial coinfection in hospitalized COVID-19 patients had small population sizes [8,9,10,11,12,14,31,32,34,35,36].

Falcone et al., performed a study to identify predictors of superinfection in COVID-19 [8]. This prospective, observational study included 315 hospitalized COVID-19 patients. In a multivariate analysis, predictor factors of superinfection were: intestinal colonization by carbapenem-resistant Enterobacterales (odds ratio (OR): 16.03, 95% confidence interval (CI): 6.5–39.5, *p* < 0.001)); invasive mechanical ventilation (OR: 5.6, 95% CI: 2.4–13.1, *p* < 0.001); immunomodulatory agents administration (tocilizumab/baricitinib) (OR: 5.09, 95% CI: 2.2–11.8, *p* < 0.001); C-reactive protein on admission > 7 mg/dl (OR: 3.59, 95% CI: 1.7–7.7, *p* = 0.001); and previous treatment with piperacillin/tazobactam (OR: 2.85, 95% CI: 1.1–7.2, *p* = 0.028) [8].

In a retrospective observational study, Thoma et al., reported a carbapenem-resistant *A. baumannii* outbreak comprising seven COVID-19 patients and identified five other carbapenem-resistant *A. baumannii* outbreak reports, with an overall patient mortality of 35% [9].

Nori et al., reported that during the COVID-19 pandemic in New York, 99 out of 152 (65%) COVID-19 patients were admitted to intensive care units, and 112 out of 152 (74%) received mechanical ventilation [10]. In total, 91 patients (60%) had positive respiratory cultures, 82 patients (54%) had positive blood cultures, and 21 patients (14%) had both positive blood and respiratory cultures with the same or different organisms. The five most frequently isolated organisms were *S. aureus* (44%), *P. aeruginosa* (16%), *Klebsiella* spp (10%), *Enterobacter* spp (8%) and *E. coli* (4%). Moreover, 17 g-negative isolates (15%) were multidrug-resistant bacteria. Among them, six (5%) were carbapenem-resistant *Enterobacteriaceae*. Among the 82 patients with positive blood cultures, 44 patients (54%) had a central venous catheter at the time of bacteremia. The following sources of infection were determined: catheter (23%), respiratory (13%), genitourinary (9%), gastrointestinal (6%), or multiple (30%). A total of 120 (79%) patients in this study had antibiotic exposure in the 30 days preceding positive microbiology, and all the 21 patients with multidrug-resistant infections had received prior antibiotics. Overall, 149 (98%) patients received antibiotics at some point during their COVID-19 hospitalizations [10].

An observational study assessed antibiotic use in COVID-19 patients admitted to the intensive care units [11]. The authors reported that antibiotics were administered to all the 52 included patients. More precisely, 49 (94.2%) patients were given antibiotics during treatment in the hospital ward and 52 (100%) during treatment in the intensive care units. Imipenem was the most frequently used antibiotic in the intensive care units (30 cases; 57.7%), followed by ceftriaxone (28 cases; 53.8%), fluoroquinolone (22 cases, 42.3%) and piperacillin/tazobactam (17 cases; 32.7%). In 18 cases (34.6%), 3 antibiotics were given simultaneously, 2 antibiotics in 29 cases (55.8%), and in 5 cases (9.6%), only 1 antibiotic was given. The mean duration of antibiotic treatment was 12.71 days [11].

Thomsen et al., reported that culture, molecular detections and ribosomal DNA genes amplification were performed on 34 respiratory samples from COVID-19 patients in the intensive care unit. Potential pathogens were detected in 7 patients (21%) by culture, in 1 patient (3%) by molecular detections and in 17 patients (50%) by ribosomal DNA genes amplification [12].

Karolyi et al., performed a retrospective observational study including 60 COVID-19 patients admitted to intensive care units with suspected hospital-acquired pneumonia, and analyzed the spectrum of detected respiratory pathogens [14]. The authors report negative, monomicrobial and polymicrobial results in 36.7%, 35% and 28.3% of the patients, respectively. The three most frequently detected bacteria were *S. aureus* (13/60, 21.7%), *K. pneumoniae* (12/60, 20%) and *H. influenzae* (9/60, 15%) [14].

In a case–control study, Nasir et al., reported that 64% of the 50 included control patients received empirical antibiotic treatment at hospital admission [32].

In a retrospective cohort study including 553 COVID-19 patients in the intensive care unit, Pourajam et al., reported an 11.9% prevalence of bacterial infection, with 55.4% of the patients receiving antibiotic treatment on admission [34].

Rebold et al., performed a retrospective cohort study including 595 COVID-19 patients, reporting a 4.2% prevalence of bloodstream infection. In this study, 80% of the patients received empirical antibiotic treatment for a median of 10 days [35].

In a multicenter, retrospective cohort study including 568 COVID-19 patients in the intensive care unit, Rouzé et al., reported a 9.7% prevalence of bacterial infection [36].

Finally, in a single-center, observational study including 266 elderly patients admitted to the department of medicine of a university hospital, Bilan et al., reported a bacterial coinfection rate of 43% [31]. Bacterial coinfection in the elderly patients was associated with longer length of stay (23 vs. 18 days, *p* =  0.026) and 30-day mortality (55.7 vs. 34.9%, *p* =  0.006) [31].

##### Benefits of Antibiotic Use in Hospitalized COVID-19 Patients

In a multicenter retrospective cohort study of older patients with COVID-19, Odille et al., included 124 hospitalized COVID-19 patients aged 75 years during the first wave of the COVID-19 pandemic [15]. The crude mortality one month after admission was compared between patients with and without antibiotic treatment. In this study, patients with antibiotics had more severe presentations. The antibiotic regimens included third-generation cephalosporins (75 patients), macrolides (50 patients), penicillin plus beta-lactamase inhibitor (40 patients) and fluoroquinolones (9 patients). Mortality rates did not significantly differ between the 2 groups at 1 month (36% of deaths in both groups). Moreover, the median duration of hospital stay was not significantly different between the 2 groups (11 vs. 10 days, *p* = 0.8) [15].

This finding was confirmed by a retrospective observational cohort study to evaluate the beneficial effect of any antibiotic administration on patients’ outcomes [16]. This cohort study included 618 hospitalized COVID-19 patients from 18 COVID-19 Italian centers, with an overall in-hospital cumulative mortality incidence of 23.1%. The multivariable models did not disclose a significant association between any single drug on the clinical outcomes.

##### Macrolides

Azithromycin has been proposed as a treatment for COVID-19 because of its immunomodulatory actions. The large RECOVERY trial evaluated the safety and efficacy of azithromycin in patients admitted to hospital with COVID-19 [17]. This was a randomized, controlled, open-label, adaptive platform trial at 176 hospitals in the UK. Patients were randomly allocated to either the usual standard of care alone or the usual standard of care plus azithromycin 500 mg once per day by mouth or intravenously for 10 days or until discharge. Among the 16,442 patients enrolled in the RECOVERY trial, 7763 were included in the assessment of azithromycin. A total of 2582 patients were randomly allocated to receive azithromycin, and 5181 patients were randomly allocated to usual care alone. The primary outcome was 28-day all-cause mortality. Overall, 561 (22%) patients allocated to azithromycin and 1162 (22%) patients allocated to usual care died within 28 days (rate ratio: 0.97, 95% CI: 0.87–1.07; *p* = 0.50). No significant difference was seen in the duration of hospital stay or the proportion of patients discharged from hospital alive within 28 days. The authors concluded that in patients admitted to hospital with COVID-19, azithromycin did not improve survival or other prespecified clinical outcomes.

The findings from the large RECOVERY trial confirmed several previous studies that found modest or no benefit in azithromycin use in patients admitted to hospital with COVID-19 [18,19,20,21,22,23,24,25,26,27].

##### Teicoplanin

Teicoplanin has been proposed in the treatment of COVID-19 pneumonia because of its potential antiviral activity against SARS-CoV-2. In a multicenter, retrospective, observational study, Ceccarelli et al., evaluated the administration of teicoplanin in the course of COVID-19 in critically ill patients [28]. This study retrospectively analyzed 55 severe COVID-19 patients hospitalized in the intensive care unit. Among them, 34 patients were treated with teicoplanin and 21 were treated without teicoplanin (control group). The authors reported that crude in-hospital mortality at 30 days was lower in the teicoplanin group (35.2%) than in the control group (42.8%). This result did not reach statistical significance (*p* = 0.654). No statistically significant differences in length of stay in the intensive care unit and in viral clearance were observed. In this study, the use of teicoplanin was not associated with a significant improvement in clinical outcomes.

##### Side Effects of Antibiotics Administration in Hospitalized COVID-19 Patients

Diarrhea is one of the manifestations of COVID-19, but it also develops as a complication of antibiotic administration. A cohort study by Maslennikov et al., aimed to characterize types of diarrhea in COVID-19 patients [29]. In this cohort study there were 89 (9.3%) patients with early viral diarrhea and 161 (16.7%) patients with late antibiotic-associated diarrhea (731 patients had no diarrhea). *Clostridioides difficile* infection was found in 70.5% of tested patients with late diarrhea and in none with early diarrhea. Presence of late diarrhea was associated with an increased risk of death after 20 days of disease (*p* = 0.009). Of importance, oral amoxicillin/clavulanate (OR: 2.23) and oral clarithromycin (OR: 3.79) use were risk factors for the development of late diarrhea.

An observational, retrospective, multicenter, 1:3 case–control study was performed to assess the incidence, outcomes and risk factors for *Clostridioides difficile* infections in COVID-19 patients [30]. During this study, 8402 COVID-19 patients were admitted to eight Italian hospitals and 32 hospital-onset *Clostridioides difficile* infections were identified. The overall incidence of hospital-onset *Clostridioides difficile* infections was 4.4 per 10,000 patient-days. In-hospital stayswas longer among cases, 35.0 vs. 19.4 days (*p* = 0.0007). Importantly, a multivariate analysis identified the administration of antibiotics during the hospital stay (*p* = 0.004) as a risk factor associated with *Clostridioides difficile* infection occurrence in COVID-19 patients [30].

#### 3.2.2. COVID-19 Outpatients

##### Bacterial Coinfection and Benefit of Antibiotic Use in COVID-19 Patients in the Community

Azithromycin

Recently, a large clinical trial (PRINCIPLE) evaluated the use of azithromycin to treat COVID-19 patients in the community [37]. The trial enrolled 2265 participants: 540 to azithromycin plus usual care, 875 to usual care alone and 850 to other interventions. In this large, UK-based, primary care, open-label, multi-arm, adaptive platform randomized trial, COVID-19 patients aged 65 years and older, or 50 years and older with at least 1 comorbidity, were randomized to usual care plus azithromycin 500 mg daily for 3 days, usual care plus other interventions, or usual care alone. In this trial, 16 (3%) of 500 participants in the azithromycin plus usual care group, and 28 (3%) of 823 participants in the usual care alone group were hospitalized. No benefit was observed. No deaths were reported in the two study groups [37].

Moreover, the “ATOMIC2” trial evaluated the efficacy of azithromycin in reducing hospital admission in patients with mild to moderate COVID-19 [38]. This was a prospective, open-label, randomized superiority trial performed at 19 hospitals in the UK. Adult COVID-19 patients were randomly assigned (1:1) to azithromycin (500 mg once daily orally for 14 days) plus standard care or to standard care alone. The primary outcome was death or hospital admission at 28 days. Among the 292 included participants, 145 were assigned to the azithromycin group and 147 to the standard care group. Overall, 15 (10%) participants in the azithromycin group and 17 (12%) in the standard care group were admitted to hospital or died during the study (OR: 0.91, 95% CI: 0.43–1.92, *p* = 0.80). Adding azithromycin to standard care treatment did not reduce the risk of subsequent hospital admission or death [38].

Finally, a randomized, double-blind clinical trial among COVID-19 outpatients was performed in the US to determine whether oral azithromycin leads to the absence of self-reported COVID-19 symptoms at two weeks [39]. The trial remotely enrolled 263 outpatients via internet-based surveys. Participants were randomized in a 2:1 fashion to a single oral 1200 mg dose of azithromycin (171 patients) or matching placebo (92 patients). This trial was terminated by the data and safety monitoring committee for futility after the interim analysis. On Day 14, there was no significant difference in proportion of participants who were symptom free (azithromycin: 50%; placebo: 50%). This trial did not support the routine use of azithromycin for COVID-19 outpatients [39].

##### Doxycicline

The PRINCIPLE trial also evaluated the efficacy of doxycycline to treat suspected COVID-19 in the community among people at high risk of adverse outcomes [40]. It included people aged 65 years or older, or 50 years or older with comorbidities (immunodeficiency, heart disease, hypertension, asthma or lung disease, diabetes, hepatic impairment, neurological problem, obesity) with suspected or confirmed COVID-19. The 1792 participants were randomly assigned to usual care only or usual care plus oral doxycycline (200 mg on Day 1, then 100 mg once daily for the following 6 days). The trial was stopped for futility. Hospitalization or death related to COVID-19 occurred in 41 (5.3%) participants in the usual care plus doxycycline group and 43 (4.5%) in the usual care-only group (estimated absolute percentage difference: −0.5%, 95% CI: −2.6 to 1.4) [40].

## 4. Guidelines on Antibiotics Use in COVID-19 Patients

A summary description of the recommendation on antibiotic use in COVID-19 from the World Health Organization, the National Institutes of Health of the United States, the European Centre for Disease Prevention and Control, the United Kingdom National Institute for Health and Care Excellence and the Italian Societies of Anti-infective Therapy and Pulmonology is reported in Table 4.

Overall, the guidelines suggest a more restrictive use of antibacterial drugs in patients with COVID-19 than that reported in the included studies [41,42,43,44,45]. In patients with severe COVID-19, the guidelines from the World Health Organization (WHO) recommend the use, as soon as possible, of empiric antimicrobials to treat all likely pathogens, based on clinical judgment, patient host factors and local epidemiology, ideally with blood cultures obtained first. Moreover, in older people, particularly those in long-term care facilities, the WHO guidelines recommend considering empiric antibiotic treatment for possible pneumonia.

In contrast, the guidelines summarized in Table 4 suggest a more restrictive use of antibiotic administration only in the presence of suspected or confirmed bacterial coinfection or secondary bacterial infections.

## 5. Discussion

The issue of bacterial infections in COVID-19 patients is receiving increasing attention. In our systematic review to assess the available evidence on the use of antibiotics in COVID-19 inpatients and outpatients, we collected data depicting widespread use of antibiotics.

At the same time, in the included studies with the largest study populations, bacterial coinfections during COVID-19 were reported in limited numbers outside the intensive care unit. Data showed that bacterial coinfection at the time of COVID-19 diagnosis is relatively uncommon, and few patients develop superinfections during hospitalization [6,7,10,13].

It should be emphasized that the included studies were extremely heterogeneous, with studies being performed in different settings with different adherence to infection prevention and control measures and antimicrobial stewardship principles.

Additionally, the way patients who developed bacterial infection were identified differed widely, with some studies reporting little information on the methods used. Not assessing the risk of bias of the included studies is an important limitation of our study.

Acknowledging these pitfalls, our finding mitigates fears that the pandemic could cause an alarming increase in the incidence of bacterial infections. Further studies carried out with more reliable and homogeneous methods are needed to confirm our preliminary finding.

Regarding the use of macrolides in hospitalized COVID-19 patients, there is strong evidence that the risk of death is not reduced by azithromycin administration [17]. Similarly, in COVID-19 patients in the community, large clinical trials found that routine antibiotic treatment with either azithromycin or doxycycline is not associated with improved outcomes [37,40].

This implies that in patients with COVID-19, antibiotic administration should be carefully evaluated. Reducing the current overuse of antibiotics may have the potential to control antibiotic resistance and antibiotic side effects, such as *Clostridioides difficile* infection, during the COVID-19 pandemic.

Recently, a large observational cohort study was performed to describe the current incidence and risk factors for bacterial coinfection in hospitalized COVID-19 patients [46]. Among 1125 consecutive COVID-19 hospitalized patients, multivariate analysis identified oxygen saturation ≤ 94% (OR: 2.47, CI: 1.57–3.86), ferritin levels < 338 ng/mL (OR: 2.63, CI: 1.69–4.07), and procalcitonin higher than 0.2 ng/mL (OR: 1.74, CI: 1.11–2.72) as independent risk factors for coinfection at hospital admission. These results suggest that empirical antimicrobial treatment may not be necessary for all patients presenting with COVID-19 infection, although the decision could be guided by high inflammatory markers and other clinical parameters [8,46].

On the other hand, the use of antibiotics, in particular high-risk groups of COVID-19 patients, should be considered. Superadded bacterial infection in older adults may be common and require treatment. An observational cohort study on elderly COVID-19 patients found high rates of superadded bacterial infection and increased length of stay and mortality, up to 45% [33]. These findings suggest adopting a more permissive antibiotic use in high-risk subgroups of COVID-19 patients, i.e., elderly patients, hematologic patients, patients receiving immunosuppression after solid organ transplantation, and patients with impairment of humoral immunity.

When we reviewed the most recent guidelines on the management of COVID-19, we observed that these documents recommend a restrictive use of antibacterial drugs in patients with COVID-19.

In patients with severe COVID-19, WHO guidelines recommend the use of empiric antimicrobials to treat all likely pathogens as soon as possible, based on clinical judgment, patient host factors and local epidemiology, ideally with blood cultures obtained first. Moreover, in older people, particularly those in long-term care facilities, the WHO guidelines recommend considering empiric antibiotic treatment for possible pneumonia. These recommendations slightly conflict with other guidelines (Table 4) that recommend antibiotic administration only in the presence of suspected or confirmed bacterial coinfection or secondary bacterial infections. All guidelines conflict with the wide use of antibiotics observed in practice.

Interestingly, the findings on the prevalence of bacterial coinfection and superinfection during SARS-CoV-2 infection are different when compared to those of other previous viral pandemics. COVID-19 seems to affect microbiological and clinical features of hospital-associated pneumonia, as well as being associated with a peculiar lung microbiota composition [47].

In the future, the development of dedicated prediction models of bacterial infection in hospitalized COVID-19 patients could help in identifying subgroups of patients that should receive empirical antibiotic treatment. These tools will have important antibiotic stewardship implications, as inappropriate use of antibiotics leads to increased antimicrobial resistance. It is likely that the overall future scenario may change from “antibiotics in COVID-19 patients are not recommended in the absence of a proven bacterial infection” to “consider antibiotics administration in high-risk COVID-19 patients”, based on the results of prediction models and the patient’s characteristic and comorbidities.

Currently, considering the risk of antimicrobial resistance and other antibiotic-related side effects, i.e., *Clostridioides difficile* infections, antibiotics should not be used for treating COVID-19 inpatients outside well-designed randomized clinical trials. Antibiotics should not be prescribed at home unless there is a strong clinical suspicion of a bacterial superinfection during COVID-19, as evidenced by the reappearance of fever, or radiological evidence of new-onset pneumonia or microbiological evidence of bacterial infection.

## Figures and Tables

**Table 1:** Summary description of the randomized clinical trials on antibiotic use in COVID-19 inpatients included in the systematic review. M: male sex.

Author, Year and Country	Study Type	Setting	Study Population, Age (Mean), Sex (% Male)	COVID-19 Severity	Study Aim	Study Design	Study Results
Rashad A et al., 2020, Egypt [18]	Phase III, double-blinded, randomized clinical trial	A single clinical site in Egypt, from May 2020 to September 2020	305 confirmed mild COVID-19 patients, Age: 44.3, Sex (M): 70%	Mild COVID-19 infection	To assess superiority of azithromycin or clarithromycin plus the standard of care in patients with mild COVID-19	Patients were randomized to three study arms: Azithromycin 500 mg/24 h for 7 days, Clarithromycin 500 mg/12 h for 7 days, or the control group	No benefit was observed in overall survival. The follow-up period was only 7 days
RECOVERY Collaborative Group. 2020, UK [17]	Randomized, controlled, open-label, adaptive platform trial	176 hospitals in the UK, Between April and November 2020	7763 COVID-19 patients, Age: 65.3, Sex (M): 62%	Mild COVID-19 infection	To evaluate the safety and efficacy of azithromycin in hospitalized COVID-19 patients	Patients were randomized to usual standard of care alone or usual standard of care plus azithromycin, 500 mg once per day for 10 days or until discharge	No significant difference was found in length of hospital stay or in mortality rate at 28 days
Furtado R et al., 2020, Brazil [20]	Open-label, randomized clinical trial	57 centers in Brazil, from March to May 2020	447 Severe COVID-19 patients, Age: 59.8, Sex (M): 65.9%	Severe COVID-19 infection	To assess the efficacy of azithromycin in improving clinical outcomes of COVID-19 patients	Patients were randomized 1:1 to azithromycin, 500 mg once daily for 10 days plus standard of care or to the standard of care alone, including hydroxychloroquine	No improvement in clinical outcomes was found
Sivapalan P et al., 2020, Denmark [24]	Randomized, double-blinded, placebo-controlled trial	6 hospitals in Denmark, between April and December 2020	117 hospitalized COVID-19 patients, Age: 65, Sex (M): 65%	Mild COVID-19 infection	To assess the efficacy of azithromycin and hydroxychloroquine in improving survival and length of hospitalization	The intervention arm received hydroxychloroquine, 200 mg twice daily for 15 days plus azithromycin, 500 mg daily for 3 days followed by 250 mg daily for 12 days. The control arm received placebo/placebo	The azithromycin and hydroxychloroquine combination did not improve survival or length of stay

**Table 2:** Summary description of the randomized clinical trials on antibiotic use in COVID-19 outpatients included in the systematic review. M: male sex.

Author, Year and Country	Study Type	Setting	Study Population, Age (Mean), Sex (% Male)	Study Aim	Study Design	Study Results
Butler C et al., 2020, UK [40]	Open-label, multi-arm, adaptive platform randomized clinical trial	Primary centers across the UK, from July to December 2020	1792 suspected COVID-19 community patients, Age: 61.1, Sex (M): 44.1%	To assess the efficacy of doxycycline in high-risk COVID-19 patients	Participants randomized to usual care alone or usual care plus oral doxycycline, 200 mg on Day 1, then 100 mg once daily for 6 days	No improvement in recovery time, hospital admission rate or COVID-19 related mortality
PRINCIPLE Trial Collaborative Group. 2020, UK [37]	Open-label, multi-arm, adaptive platform randomized clinical trial	Primary centers across the UK, from May to November 2020	2120 suspected COVID-19 patients, Age: 60.7, Sex (M): 43%	To assess the efficacy of azithromycin in high-risk COVID-19 patients	Participants randomized to usual care alone or usual care plus azithromycin, 500 mg daily for 3 days	No significant improvement in recovery time
Hinks T et al., 2020, UK [38]	Prospective, open-label, randomized superiority clinical trial	19 hospitals in the UK, from June 2020 to January 2021	298 community patients with mild to moderate COVID-19, Age: 45.9, Sex (M): 51.5%	To evaluate azithromycin efficacy in reducing hospital admission rate	Participants were randomized 1:1 to azithromycin, 500 mg daily for 14 days plus standard of care or standard of care alone	No improvement in mortality or in-hospital admission rate
Oldenburg C et al., 2020, US [39]	Randomized, placebo-controlled trial	The US, from May 2020 to March 2021	263 community COVID-19 patients, Age: 42.7, Sex (M): 32.7%	To assess azithromycin efficacy in reducing self-reported COVID-19 symptoms at 14 days	Participants were randomized 2:1 to azithromycin, 1200 mg single oral dose or placebo	No significant difference in the proportion of participants reporting symptoms

**Table 3:** Antibiotics use: bacterial infection microbial etiology in hospitalized COVID-19 patients.

Author, Year	Study Design	Study Population, Setting	Type of the Clinical Specimens Collected for Bacterial Detection	Bacterial Coinfection, Percentage	Empirical Antibiotic Treatment at Hospital Admission, Percentage	Microbial Etiology
Garcia-Vidal C, 2021 [5]	Observational cohort study	989 COVID-19 patients. All the hospital wards	Blood, normally sterile fluids, sputum and other samples	7.2%	Yes, more than 90% of the patients	*S. pneumoniae* *S. aureus* *P. aeruginosa* *E. coli*
Karami Z, 2020 [6]	Retrospective observational study	925 COVID-19 patients. All the hospital wards	Sputum, blood cultures, pneumococcal and *Legionella* urinary antigen tests	1.2%	Yes, 60.1% received antibiotics before hospital admission	*E. coli* *S. aureus* *Achromobacter* spp
Wang L, 2020 [7]	Retrospective observational cohort study	1396 COVID-19 patients. All the hospital wards	Nasopharyngeal or lower respiratory tract specimens	2.7%	Yes, 98% received antibiotics at admission	*E. coli* (ESBL-producing) *K. pneumoniae* *K. variicola* *P. mirabilis* MRSA MSSA *S. epidermidis* Group A *Streptococcus* *H. influenzae* *P. aeruginosa*
Falcone M, 2020 [8]	Prospective observational study	315 COVID-19 patients. All the hospital wards	Pneumonia, urinary tract infection, skin and soft structure infection, intra-abdominal and bloodstream infection	21.9%	Yes	Enterobacterales Non-fermenting Gram-negative bacilli Gram-positive bacteria Fungi
Rebold N, 2022 [35]	Retrospective cohort study	595 COVID-19 patients. All the hospital wards	Only blood	4.2% (only blood infection)	Yes, 80%	Coagulase-negative *Staphylococci* (36%) *S. aureus* (20%) *Enterococcus* (16%) Enterobacterales (8%)
Bilan J, 2022 [31]	Single-center observational study	266 older adults with COVID-19. Department of Medicine	Any clinical specimen	43%	Yes	*S. haemolyticus* *S. aureus* *P. aeruginosa* *P. mirabilis* *P. acnes* *K. pneumoniae* *P. aeruginosa* *H. influenzae* *E. coli* *E. faecalis* *C. difficile*
Nasir N, 2021 [32]	Case–control study	50 cases, 50 controls. All the hospital wards	Various sites including urinary tract, lungs, blood	100% cases, N/A controls	Yes (cases: 100%, controls: 64%)	*Acinetobacter* spp. (22%) *P. aeruginosa* (21%) *S. maltophilia* (13%) *S. aureus* (10%) *K. penumoniae* (8%) *E. coli* (3%)
Giannella M, 2022 [33]	Multicenter observational study	1733 hospitalized COVID-19 patients. All the hospital wards	Any clinical specimen	6.3%	Yes, 59.8%	*Enterobacteriaceae* (33.6%) *S. pneumoniae* (29.1%) *E. Coli* (28.2%) Coagulase-negative *Staphylococci* (10%) *M. pneumoniae* (8.2%) *K. pneumoniae* (6.4%) Others (19%)
Ruiz-Bastián M, 2021 [13]	Retrospective observational cohort study	1195 COVID-19 patients. Clinical Microbiology and Parasitology Department	Respiratory samples including bronchial aspirates, bronchoalveolar lavages and tracheal aspirates	5.52%	Not assessed	Enterobacterales *S. aureus* *P. aeruginosa*
Karolyi M, 2022 [14]	Retrospective observational cohort study	60 COVID-19 patients. Infectious Diseases Department	Respiratory samples	35% (monomicrobial) 28.3% (polymicrobial)	Yes, 73% of the patients	*S. aureus* (21.7%) *K. pneumoniae* (20%) *H. influenzae* (15%)
Nori P, 2020 [10]	Retrospective observational cohort study	4267 COVID-19 patients. Hospital wards, including intensive care unit	Blood or respiratory samples	3.6%	Yes, 71%	*S. aureus* (30%) *S. epidermidis* (12%) *Streptococcus* spp. (10%) *Enterococcus* spp. (7%) *E. coli* (7%) *P. aeruginosa* (6%) *Candida* spp. (5%) *Klebsiella* spp. (3%) *Enterobacter* spp. (3%)
Thoma R, 2022 [9]	Retrospective observational cohort study	10 COVID-19 patients. Intensive care unit	Rectum, skin, urine, respiratory tract	100%	Not assessed	Carbapenem-resistant *A. baumannii*
Mustafa L, 2021 [11]	Retrospective observational cohort study	52 COVID-19 patients. Intensive care unit	Nasopharyngeal swabs	Not assessed	Yes, 94%	Not reported
Thomsen K, 2021 [12]	Observational cohort study	34 COVID-19 patients. Intensive care unit	Lower respiratory samples	3–21%	Yes, 59%	*S. aureus* *S. pneumoniae*
Pourajam S, 2022 [34]	Retrospective cohort study	553 COVID-19 patients. Intensive care unit	Blood, cerebrospinal fluid, sputum, stool, tracheal aspirate, wound and urine	11.9%	Yes, 55.4%	*K. pneumoniae* (72.3%) *A. baumannii* (53.8%) *E. cloacae* (1.5%) *E. coli* (1.5%) *P. aeruginosa* (1.5%)
Rouzé A, 2021 [36]	Multicenter retrospective cohort study	568 COVID-19 patients. Intensive care unit	Endotracheal aspirates, blood, pneumococcal or *Legionella* urinary antigen test	9.7%	Yes (88% at intensive care unit admission)	*S. aureus* (23.6%) *S. pneumoniae* (21.8) *P. aeruginosa* (10.9%) *H. influenzae* (9.1%) *Streptococcus* spp (7.3%) Other (40%)

ESBL: Extended-spectrum beta-lactamases; MSSA: Methicillin-Sensitive *Staphylococcus aureus*; MRSA: Methicillin-Resistant *Staphylococcus aureus*.

**Table 4:** Summary description of the clinical guidelines’ main recommendations on antibiotic treatment in COVID-19 inpatients and outpatients.

Guideline	Last Update	General Recommendations on Antibiotic Treatment	COVID-19 Inpatients	COVID-19 Outpatients
World Health Organization (WHO) [41]	November 2021	Antibiotic therapy not recommended in patients with mild COVID-19. In patients with moderate COVID-19 antibiotics should not be prescribed unless a bacterial infection is suspected	In patients with severe COVID-19, the guideline recommends empiric antibiotic treatment, based on clinical judgment, patient host factors and local epidemiology, as soon as possible	Guideline recommends considering empiric antibiotic treatment in the elderly, particularly in long-term care facility setting
National Institutes of Health (US) [42]	May 2022	Empiric antibiotic treatment is not recommended	Guideline recommends following the guidelines established for non-COVID-19 patients	Antibiotic treatment is not recommended
The European Centre for Disease Prevention and Control (Europe) [43]	February 2022	Consider antibiotic treatment only if bacterial coinfection is suspected/confirmed	Routine azithromycin administration is not recommended	Antibiotic treatment only if bacterial coinfection is suspected/confirmed
The National Institute for Health and Care Excellence (UK) [44]	April 2022	Consider antibiotic administration only if bacterial coinfection is suspected or confirmed	Consider antibiotic administration only if bacterial coinfection is suspected or confirmed	Doxycycline is not recommended
Italian Society of Anti-infective Therapy and Italian Society of Pulmonology (Italy) [45]	July 2021	Antibiotic administration is not recommended in the absence of a proven bacterial infection	Consider empirical antibiotic treatment if radiological signs of pulmonary consolidative lesions. Collection of samples for culture or molecular detection before antibiotic administration is recommended	Azithromycin is not recommended

## Data Availability

The data presented in this study are openly available in the MEDLINE and SCOPUS repository.

## Supplementary Materials

The following supporting information can be downloaded at: https://www.mdpi.com/article/10.3390/jcm11237207/s1, Figure S1: The complete specifications of the queries used to search the MEDLINE and SCOPUS database for the systematic review.
