# Supplementary material for: Antibiotics Use in COVID-19 Patients: A Systematic Literature Review
Source: J Clin Med. 2022 Dec 4;11(23):7207. doi: 10.3390/jcm11237207 (PMC9739751; doi:10.3390/jcm11237207)
Supplement: Supplementary file 1 [file jcm-11-07207-s001.zip › jcm-1981421-supplementary.pdf]

Supplementary Figure S1. The complete specifications of the queries used to search the MEDLINE and SCOPUS database for the systematic review.

MEDLINE query:

Search: Antibiotic AND COVID-19 Filters: Clinical Study, Clinical Trial, Observational Study, Randomized Controlled Trial, English, Adult: 19+ years, from 2020/1/1 - 2022/10/1 Sort by: First Author

(("anti bacterial agents"[Pharmacological Action] OR "anti bacterial agents"[MeSH Terms] OR ("anti bacterial"[All Fields] AND "agents"[All Fields]) OR "anti bacterial agents"[All Fields] OR "antibiotic"[All Fields] OR "antibiotics"[All Fields] OR "antibiotic s"[All Fields] OR "antibiotical"[All Fields]) AND ("covid 19"[All Fields] OR "covid 19"[MeSH Terms] OR "covid 19 vaccines"[All Fields] OR "covid 19 vaccines"[MeSH Terms] OR "covid 19 serotherapy"[All Fields] OR "covid 19 serotherapy"[Supplementary Concept] OR "covid 19 nucleic acid testing"[All Fields] OR "covid 19 nucleic acid testing"[MeSH Terms] OR "covid 19 serological testing"[All Fields] OR "covid 19 serological testing"[MeSH Terms] OR "covid 19 testing"[All Fields] OR "covid 19 testing"[MeSH Terms] OR "sars cov 2"[All Fields] OR "sars cov 2"[MeSH Terms] OR "severe acute respiratory syndrome coronavirus 2"[All Fields] OR "ncov"[All Fields] OR "2019 ncov"[All Fields] OR (("coronavirus"[MeSH Terms] OR "coronavirus"[All Fields] OR "cov"[All Fields]) AND 2019/11/01:3000/12/31[Date - Publication]))) AND ((clinicalstudy[Filter] OR clinicaltrial[Filter] OR observationalstudy[Filter] OR randomizedcontrolledtrial[Filter]) AND (2020/1/1:2022/10/1[pdat]) AND (english[Filter]) AND (alladult[Filter]))

Translations

Antibiotic: "anti-bacterial agents"[Pharmacological Action] OR "anti-bacterial agents"[MeSH Terms] OR ("anti-bacterial"[All Fields] AND "agents"[All Fields]) OR "anti-bacterial agents"[All Fields] OR "antibiotic"[All Fields] OR "antibiotics"[All Fields] OR "antibiotic's"[All Fields] OR "antibiotical"[All Fields]

COVID-19: ("COVID-19" OR "COVID-19"[MeSH Terms] OR "COVID-19 Vaccines" OR "COVID-19 Vaccines"[MeSH Terms] OR "COVID-19 serotherapy" OR "COVID-19 serotherapy"[Supplementary Concept] OR "COVID-19 Nucleic Acid Testing" OR "covid-19 nucleic acid testing"[MeSH Terms] OR "COVID-19 Serological Testing" OR "covid-19 serological testing"[MeSH Terms] OR "COVID-19 Testing" OR "covid-19 testing"[MeSH Terms] OR "SARS-CoV-2" OR "sars-cov-2"[MeSH Terms] OR "Severe Acute Respiratory Syndrome Coronavirus 2" OR "NCOV" OR "2019 NCOV" OR (("coronavirus"[MeSH Terms] OR "coronavirus" OR "COV") AND 2019/11/01[PDAT] : 3000/12/31[PDAT]))

SCOPUS query:

ALL (antibiotic AND treatment AND covid-19) AND PUBYEAR > 2019 AND PUBYEAR < 2023 AND (LIMIT-TO (EXACTKEYWORD , "Outcome Assessment")) AND (LIMIT-TO (SUBJAREA , "MEDI")) AND (LIMIT-TO (DOCTYPE , "ar")) AND (LIMIT-TO (LANGUAGE , "English"))
